# Human induced pluripotent stem cell-derived cardiac myocytes and sympathetic neurons in disease modelling

**DOI:** 10.1098/rstb.2022.0173

**Published:** 2023-06-19

**Authors:** Ni Li, Michael Edel, Kun Liu, Chris Denning, Jacob Betts, Oliver C. Neely, Dan Li, David J. Paterson

**Affiliations:** ^1^ Burdon Sanderson Cardiac Science Centre and BHF Centre of Research Excellence, Department of Physiology, Anatomy and Genetics, University of Oxford, Oxford OX1 3PT, UK; ^2^ Nuffield Department of Medicine, Chinese Academy of Medical Sciences Oxford Institute, Oxford OX3 7BN, UK; ^3^ Faculty of Medicine, Unit of Anatomy and Embryology, Autonomous University of Barcelona, Barcelona 08193, Spain; ^4^ Discipline of Medical Sciences and Genetics, School of Biomedical Sciences, University of Western Australia, Perth 6009, Australia; ^5^ Faculty of Medicine & Health Sciences, University of Nottingham Biodiscovery Institute, Nottingham NG7 2RD, UK

**Keywords:** hiPSC, cardiac myocytes, sympathetic neurons, neurocardiac co-culture

## Abstract

Human induced pluripotent stem cells (hiPSC) offer an unprecedented opportunity to generate model systems that facilitate a mechanistic understanding of human disease. Current differentiation protocols are capable of generating cardiac myocytes (hiPSC-CM) and sympathetic neurons (hiPSC-SN). However, the ability of hiPSC-derived neurocardiac co-culture systems to replicate the human phenotype in disease modelling is still in its infancy. Here, we adapted current methods for efficient and replicable induction of hiPSC-CM and hiPSC-SN. Expression of cell-type-specific proteins were confirmed by flow cytometry and immunofluorescence staining. The utility of healthy hiPSC-CM was tested with pressor agents to develop a model of cardiac hypertrophy. Treatment with angiotensin II (AngII) resulted in: (i) cell and nuclear enlargement, (ii) enhanced fetal gene expression, and (iii) FRET-activated cAMP responses to adrenergic stimulation. AngII or KCl increased intracellular calcium transients in hiPSC-SN. Immunostaining in neurocardiac co-cultures demonstrated anatomical innervation to myocytes, where myocyte cytosolic cAMP responses were enhanced by forskolin compared with monocultures. In conclusion, human iPSC-derived cardiac myocytes and sympathetic neurons replicated many features of the anatomy and (patho)physiology of these cells, where co-culture preparations behaved in a manner that mimicked key physiological responses seen in other mammalian systems.

This article is part of the theme issue ‘The heartbeat: its molecular basis and physiological mechanisms’.

## 1. Introduction

Stem cell science gained momentum when James Thomson in 1998 [1] grew the first embryonic stem cells in a dish. This set the platform for Christine Mummery in 2001 [2] to use stem cells to create beating heart cells outside the body. However, it was the reprogramming of fibroblasts back to their pluripotent state in 2006 by Takahashi & Yamanaka that resulted in human induced pluripotent stem cell technology transforming biology and providing an opportunity to study human disease *in vitro* [3,4]. In heart physiology, the induction of cardiomyocytes from human induced pluripotent stem cells (hiPSC) was rapidly realized by applying the standard embryoid bodies-based protocol for embryonic stem cells [5,6], which was optimized over time by more precise control of the embryonic development signal [7–10]. To date, the speed to generate hiPSC has been enhanced and genetic stability improved using novel modified synthetic mRNA transfection methods [11,12].

While primary cells from patients with genetic-based arrhythmias are largely unavailable, hiPSC has emerged as a tool to model inherited cardiac syndromes [13–16]. Human iPSC-induced cardiomyocytes were first applied in a mechanistic study of long-QT syndrome (LQTS) in 2010 [13], where patient-derived cells were capable of replicating the electrical phenotype of the disease. Given the power of the sympathetic nervous system to trigger arrhythmia in patients with cardiac channelopathies [17,18], attention has recently focused on developing neuronal–cardiac co-culture systems with either animal cells or stem cells in order to facilitate the study of neurocardiac interactions [19–25].

This has met with some success. Oh *et al*. [21] were first to report evidence for functional coupling, where noradrenaline-secreting sympathetic neurons in co-culture were capable of driving heart rate in neonatal cardiac myocytes when stimulated pharmacologically or optogenetically. Similarly, others [23,24] showed selective induction of healthy human sympathetic neurons could enable precise control of cardiac myocyte beating. Development of a diseased cardiac–neural co-culture system has not been firmly established, although Winbo *et al*. [26] recently reported that sympathetic-derived neurons in monoculture from an LQT1 patient (*KCNQ1* loss-of-function variants) showed increased excitability and neurotransmission. Whether an hiPSC cardiac–neural co-culture system can also replicate and mimic cardiac–neural responses seen in other mammalian systems remains to be established.

Here we adapted current protocols of cardiac myocytes and sympathetic neuronal differentiation from hiPSC and conducted physiological phenotyping of these cells to confirm cell type and cell function. Simulation of a human disease was tested by exposure of hiPSC-cardiac myocytes (hiPSC-CM) to pressor agents to induce hypertrophy. We also developed hiPSC-sympathetic neurons (hiPSC-SN) in mono-culture and co-culture with hiPSC-CM, and assessed the utility of these cultures to respond to physiological stimuli.

## 2. Methods

### (a) hiPSC culture

The two healthy hiPSC lines (SFC854-03-02 and iPS-OX1-19) used for cardiac myocyte differentiation in this study have been published previously [27,28]. Lines were derived from dermal fibroblasts from healthy donors through StemBANCC (SFC854-03-02) or the Oxford Parkinson's Disease Centre (iPS-OX1-19): participants were recruited to this study having given signed informed consent, which included derivation of hiPSC lines from skin biopsies (Ethics Committee that specifically approved this part of the study: for control donors, National Health Service, Health Research Authority, NRES Committee South Central, Berkshire, UK, REC 10/H0505/71); all experiments were performed in accordance with UK guidelines and regulations and as set out by the Research Ethics Committee (REC). Three cell lines, iPS-OX1-19, UKKi007-A and ReBl-PAT^RYR^^2-WT^, were used for sympathetic neuron induction. UKKi007-A was provided by the European Collection of Authenticated Cell Cultures, and ethically approved by the Ethics Committee of the Ruhr-University Bochum, Bad Oeynhausen, Germany, registration no. 41/2008. ReBl-PAT^RYR2-WT^ isolation and use of patient fibroblasts were approved by the Nottingham Research Ethics Committee (License 09/H0408/74) and sample collections are registered with the UK Clinical Research Network under project 8164. hiPSC were amplified in Matrigel-coated (Corning, 356234) cell culture flasks in mTeSR medium (STEMCELL Technologies, 85850), then were frozen at 2 × 10^6^ cells ml^−1^ in Cryostor (STEMCELL Technologies, 07959) in liquid nitrogen for long-term storage. Frozen hiPSC were thawed and passaged three times for amplification and revitalization before differentiation commenced. For protocol reproducibility, both cardiomyocyte and sympathetic neuron induction were repeated more than three times on SFC854-03-02 and iPS-OX1-19 lines.

### (b) hiPSC-CM differentiation

**Figure 1.**
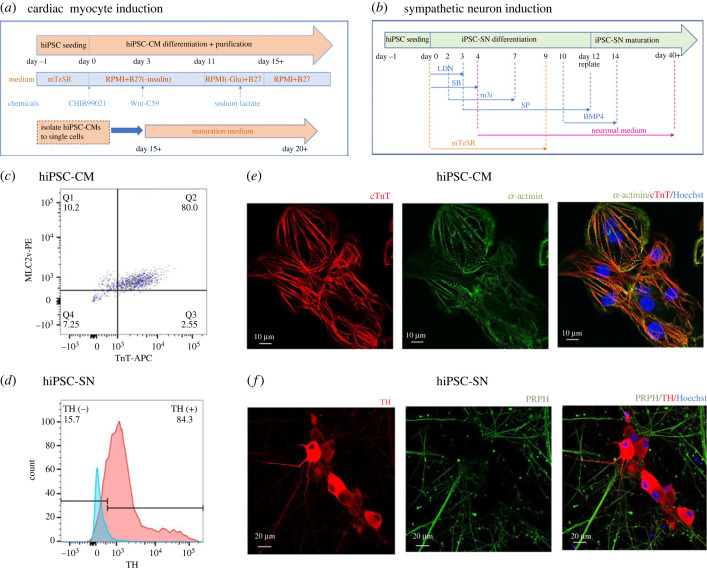
Induction of cardiac myocytes and sympathetic neurons from human induced pluripotent stem cells. (*a,b*) Schematic summary of cardiac myocyte (*a*) and sympathetic neuron (*b*) induction protocols. (*c*,*d*) Flow cytometric results from day 20 cardiac myocytes (*c*) and day 50 sympathetic neurons (*d*). In (*c*) the right upper quadrant (Q2) shows positive population for ventricular myosin light chain 2 (MLC2v) and cardiac troponin (cTnT) in hiPSC-CM (from SFC854-03-02) of 80%. (*d*) Blue curve represents blank control and red curve represents hiPSC-SN (from UKKi007-A) stained with anti-tyrosine hydroxylase (anti-TH). Compared with negative control, 84.3% of hiPSC-SN expressed TH. (*e,f*) Structural characterization of day 20 cardiac myocytes (*e*) and day 42 sympathetic neurons (*f*) by immunofluorescence staining. The alignment of cTnT and α-actinin shows sarcomere structure within cardiac myocytes (*e*). Anti-TH-stained neuron cell body and axon; peripherin (PRPH) staining outlines the axon extension and formation of varicosities (*f*).

Thawed iPSCs were passaged three times then seeded on Matrigel-coated 12-well plates and cultured in mTeSR medium until 85–90% confluent. On day 0, the medium was changed to RPMI (Gibco, 11875093)/B27-insulin (Gibco, A1895601) (RB–) with 6 µM CHIR99021 (Tocris Bioscience, 4423) for 48 h of incubation, and exchanged for RB– on day 2. On day 3, 2.5 µM Wnt-C59 (Tocris Bioscience, 5148) was applied in RB– for 48 h. From day 5, cells were fed with RB– medium, which was changed every 2 days. Purification was conducted by changing the medium to RPMI-glucose (Gibco, 11879020)/B27 (Gibco, 17504044) plus 5 mM sodium l-lactate (Sigma, 71718) to remove the glucose nutrient supplement from day 11 to day 14. After purification, hiPSC-CM were kept in RPMI/B27 medium (RB+) from day 15. For experiments, cells were dissociated with TrypLE enzyme (Gibco, 12604013), and re-seeded on FluoroDish tissue culture dishes (WPI, FD35PDL) or coverslips on around day 20. Maturation medium (DMEM low glucose (Sigma, D5546) plus 4% oleic acid–BSA (Sigma, O3008), 10 µg ml^−1^ insulin (Sigma, I9278), 2 mM glutamine, 10% horse serum) was applied prior to experiments to enhance myocyte maturation when required (figure 1*a*).

### (c) hiPSC-SN differentiation

Sympathetic neurons were differentiated from hiPSC according to the protocol proposed by Winbo *et al*. [24]. Briefly, after three passages, hiPSC were seeded on Matrigel-coated 12-well plates and cultured in mTeSR until 80% confluent. Then a series of chemical compounds were added sequentially (500 nM LDN193189 (Sigma, SML0559) days 0–3, 10 µM SB431542 (Sigma, S4317) days 0–4, 3 µM CHIR99021 + 10 µM DAPT (Sigma, D5942) + 0.2 µM PD173074 (Sigma, P2499) days 2–7, 60 ng ml^−1^ Shh C25II (Gibco, PMC8034) + 1 µM PMP (Sigma, SML0868) days 3–12, 10 ng ml^−1^ BMP4 (R&D Systems, 314-BP-010) days 10–14) to the culture medium to modify pathways involved in the induction of neuromesodermal progenitors and neural crest cells. Culture medium mTeSR was gradually replaced by N2 medium (Neurobasal Plus medium (Gibco, A3582901) supplemented with 2 mM L-glutamine, B-27 Plus (Gibco, A3582801), N-2 supplements (Gibco, 11520536), 0.2 mM ascorbic acid (Sigma, A8960), 0.2 nM dbcAMP (Sigma, D0260), 10 ng ml^−1^ NGF (Bio-Techne, 256-GF-100), 10 ng ml^−1^ BDNF (R&D Systems, 248-BDB-010), 10 ng ml^−1^ GDNF (R&D Systems, 212-GD-010)) to promote sympathetic neuron development from day 4 (days 4–5: 75% mTeSR + 25% N2 medium; days 6–7: 50% mTeSR + 50% N2 medium; days 8–9: 25% mTeSR + 75% N2 medium; days 10–12: N2 medium). On day 12, differentiated cells were detached with Accutase (Gibco, A1110501), re-seeded on 24-well plates at a density of 10^6^ cells per well and cultured in neuronal medium for 40 days for maturation, after which characterization and co-culture with iPSC-CM was carried out (figure 1*b*).

### (d) Neurocardiac co-culture

Contracting hiPSC-CM over day 20 and hiPSC-SN over day 40 with abundant axon extensions were digested on the same day and reseeded together in Matrigel-coated culture plates for neurocardiac co-culture establishment. TrypLE enzyme was used for hiPSC-CM dissociation by incubating for 7–10 min in a 37°C CO_2_ incubator. hiPSC-SN were dissociated by 20–40 min incubation in 1 mg ml^−1^ collagenase A (Roche, 10103586001) in Hanks' balanced salt solution. Detached cells were centrifuged and resuspended in 1 : 1 RB+ plus N2 medium, and the seeding density of myocytes to neurons was 1 : 1. The medium was changed every other day.

### (e) Isolated rat ventricular myocytes

Animal use complied with the University of Oxford Local Ethical Guidelines and was in accordance with the *Guide for the care and use of laboratory animals* [29] published by the US National Institutes of Health (publication no. 85-23, revised 2011) and the Animals (Scientific Procedures) Act 1986 (United Kingdom). Experiments were performed under British Home Office Project License P707EB251 (D.J.P.). Ventricles were removed from *ca* 1–3-day-old Wistar neonatal rats in accordance with approved Schedule 1 of the Act, killing by dislocation of the neck, and digested by stirring for 20 min in collagenase (Roche, 10103586001) and pancreatin (Sigma, P1750) buffer at 37°C five times. After each 20 min stirring, supernatants containing cardiac myocytes were collected and centrifuged, and cell pellets were resuspended. After digestion, all cell suspensions were combined to spin at 1250 r.p.m. (175*g*), 5 min, 4°C. Pellets containing cardiomyocytes were resuspended in culture medium (DMEM high glucose (Invitrogen, 42430025) plus 17.5% M199 (Invitrogen, 31150022), 10% horse serum, 5% newborn calf serum, 2 mM glutamine, 0.1% penicillin/streptomycin) and seeded on cell culture dishes for 1 h to get rid of fibroblasts and endothelial cells. Unattached myocytes were collected from supernatant and plated on appropriate cell culture dishes for further experiments.

### (f) Immunofluorescence staining

Induced cardiomyocytes and sympathetic neurons from hiPSC were dissociated and seeded on FluoroDish tissue culture dishes at desirable density for subsequent confocal imaging. Two to five days after seeding, cells were fixed with 4% formaldehyde, permeabilized and blocked in Tris-buffered saline (TBS) plus 0.5% Triton and 6% donkey serum. hiPSC-CM were then incubated in TBS with primary antibodies, anti-α-actinin (Sigma, A7811) and anti-cardiac troponin T (anti-cTnT, Abcam, ab45932), overnight at 4°C. For hiPSC-SN, anti-tyrosine hydroxylase (anti-TH, Sigma, T2928), anti-peripherin (anti-PRPH, Sigma, AB1530) and anti-synapsin (anti-Syn, Abcam, ab254349) were used. Then secondary antibodies conjugated to Alexa Fluor (488 and 594) were applied at room temperature for 2 h. Cell nuclei were stained by Hoechst (Invitrogen, H3570) for 10 min. Images were taken by Leica confocal microscope (Leica TCS SP5) and analysed with LAS X software.

### (g) Flow cytometry

Differentiated cells were collected in single cell suspension and centrifuged. Pellets were resuspended and incubated with Live/Dead stain (ThermoFisher, 65-0865-14), then fixed with 4% formaldehyde, permeabilized and blocked in blocking buffer, followed by incubation with anti-cTnT (MACS 130-120-403) and anti-ventricular myosin light chain-2 (MLC2v, MACS 130-119-581) antibodies for hiPSC-CM, and anti-TH (Abcam, ab209921) for hiPSC-SN, at required concentrations. Dead cell sample, unstained sample and compensation beads were prepared as control groups. Cells were then resuspended in FACS buffer (phosphate-buffered saline (without calcium, magnesium, Corning, 21-040-CV) + 2% fetal bovine serum (Gibco, 16140071) + 0.02% NaN_3_ (Sigma, 71289)) for running in a flow machine (BD Fortessa X20). Results were analysed by FlowJo software.

### (h) Cell size and nuclear size measurement

Day 20 hiPSC-CM were re-plated on FluoroDish tissue culture dishes, and treated with AngII (MedChem Express, HY-13948A) 100 nM, 1 μM for 48 h to induce cardiac hypertrophy [30]. Then, control and hypertrophic myocytes were fixed and stained for cTnT and α-actinin. Images taken were analysed by ImageJ software. Nuclear area was automatically selected using the ImageJ wand (tracing) tool after thresholding each Hoechst-stained image layer in ImageJ to identify pixels that shape the nuclear area. The selected area was entered into ROI Manager, where pixel size could be automatically measured. Pixel size was converted to physical size in micrometres according to image scale.

### (i) Reverse transcription-quantitative polymerase chain reaction (RT-qPCR)

Total RNA from control and hypertrophic hiPSC-CM was extracted using the RNeasy mini kit (Qiagen, 74104) according to the manufacturer's instructions. Reverse transcription of extracted RNA was performed using the iScript cDNA synthesis kit (Bio-Rad, 1708891). Subsequent reverse transcription-quantitative polymerase chain reaction (RT-qPCR) was conducted with 20 µl reaction mixture composed of 2 µl cDNA plus 7 µl RNase-free water, 1 µl TaqMan Gene Expression Assay (20X) (*NPPB* Hs00173590_m1; *NPPA* Hs00383230_g1; *MYH6* Hs01101425_m1; *MYH7* Hs01110632_m1) and 10 µl TaqMan Universal PCR Master Mix (Applied Biosystems, 4304437). Amplification was conducted in QuantStudio 5 with thermal cycling parameters as follows: 10 min at 95°C, followed by 40 cycles of 15 s at 95°C and 1 min at 60°C. Amplification curves were generated automatically by the real-time PCR system. Relative quantification compared target gene expression between control and hypertrophic groups using the comparative Δ*C*_T_ method. *GAPDH* were used as internal control.

### (j) Pro-B-type natriuretic peptide (proBNP) secretion

The proBNP (precursor of active BNP that is secreted from cardiac myocytes, an indicator of ventricular injury) concentration in cardiomyocyte culture media was measured using the Human proBNP ELISA kit (Invitrogen, EHPRONPPB) according to the manufacturer's instructions. Cell culture media were collected from hypertrophic and control groups. Collected samples were transferred to the supplied proBNP antibody-coated 96-well plates in duplicate, followed by adding of biotin, streptavidin-HRP, TMB substrate (3,3',5,5'-tetramethylbenzidine, supplied in the proBNP kit) and stop solution sequentially. Absorbance at 450 nm was measured by spectrophotometer (Thermo Multiskan GO).

### (k) Förster resonance energy transfer (FRET) imaging

Cardiomyocytes were transfected with adenoviral vectors encoding the Förster resonance energy transfer (FRET) cAMP sensor Epac-S^H187^ by incubation overnight at 37°C, 5% CO_2_ prior to the FRET measurements. Cytosolic cAMP response to different stimuli in the transfected cells was recorded as cyan fluorescent protein (CFP)/yellow fluorescent protein (YFP) emission intensity change. Cells were bathed in Tyrode solution (135 mM NaCl (Sigma, S7653), 4.5 mM KCl (Sigma, P9541), 20 mM HEPES (Sigma, H3375), 11 mM glucose (Sigma, G7021), 1 mM MgCl_2_ (SERVA, 39772.02), 2 mM CaCl_2_ (BioVision, B1010), titrated with NaOH to pH 7.4) to reach a stable baseline before isoprenaline (Sigma, I6504), IBMX (non-selective phosphodiesterase inhibitor; 3-isobutyl-1-methylxanthine, Sigma, I5879) and forskolin (FSK, adenylyl cyclase agonist; Cayman Chemical, 11018) were applied. Images were taken using a Nikon Eclipse Ti2 inverted microscope with a 40× oil immersion objective, connected to a Cairn Research dual OptoLED light source, and a Prime BSI photometric camera. Excitation wavelength was set at 430 nm, and emission was measured at 480 and 535 nm for CFP and YFP, respectively. Background fluorescence was subtracted from the recorded emission intensities, and FRET ratio was calculated as CFP over YFP and normalized to the average FRET ratio at baseline for comparison.

### (l) Calcium imaging

Intracellular calcium concentration ([Ca^2+^]_i_) was measured in hiPSC-induced sympathetic neurons using Fura-2 AM live cell calcium indicator. Cells were loaded with 5 µM Fura-2 AM (Invitrogen, F1221) for 30 min at 37°C, then were washed three times prior to imaging. Throughout imaging, cells were bathed in Tyrode solution at 36.5°C, supplemented with AngII (100 or 200 nM) at 2 min and 50 mM KCl at 5 min. Images were captured at 340 and 380 nm excitation with a QIClick digital CCD camera (QImaging) connected to an OptoLED fluorescence imaging system housed on a Nikon Eclipse Ti2 inverted microscope with a 40× oil immersion objective. Calcium concentration was measured as ratio of emission triggered by 340 nm over 380 nm and expressed as fold change from the normalized baseline.

### (m) Statistical analysis

All data were processed and analysed using Prism 9.0 software. Differences between groups were assessed by *t*-test (two groups) and ANOVA (more than two groups); non-parametric *t*-test and ANOVA were used for data that were not normally distributed. Results are expressed as mean ± s.e. Significance is indicated as *p < 0.05, **p < 0.01, ***p < 0.001, ^#^*p* < 0.0001, n.s., not significant.

## 3. Results

### (a) hiPSC differentiation to cardiac myocytes and sympathetic neurons

The induction procedures of cardiac myocytes and sympathetic neurons from hiPSC are illustrated in figure 1*a,b*. Cardiomyocyte differentiation was adapted from a well-established small-molecule-modulated differentiating approach [8,31,32]. There have been several published methods reporting sympathetic neuronal differentiation from hiPSC [24,33–35]. Here we adapted the method reported by Winbo *et al*. [24].

Flow cytometry was used to validate induction efficiency of hiPSC-CM and hiPSC-SN. Day 20 hiPSC-CM were stained by anti-cTnT and anti-MLC2v. According to positive and negative signals set by compensation beads, and compared with non-stained control, 80% of hiPSC-CM induced from SFC854-03-02 hiPSC expressed both cardiac-specific proteins (figure 1*c*). Day 42 hiPSC-SN (derived from UKKi007-A) was stained by anti-TH, with 84.3% of hiPSC-SN showing positive for TH expression compared with non-stained control sample (figure 1*d*).

Cell morphology was further characterized by immunofluorescence staining. Anti-cTnT and anti-α-actinin were used for hiPSC-CM staining. cTnT is part of the troponin complex that covers the actin thin filament, spanning along the longitudinal sarcomere, and α-actinin crosslinks the thin filaments and serves as a binding point for thick filaments to the Z-disc. Staining of cTnT and α-actinin in hiPSC-CM colocalized and showed the structure of adjoining sarcomeres (figure 1*e*), establishing evidence to support the cells being cardiac myocytes. Anti-TH and anti-PRPH were used for hiPSC-SN staining. As a filamentous protein responsible for neurite elongation, PRPH labelled the axons of hiPSC-SN, and cell bodies were dominated by TH, confirming their nature as sympathetic neurons (figure 1*f*).

### (b) Pathological hypertrophy modelling using hiPSC-CM

**Figure 2.**
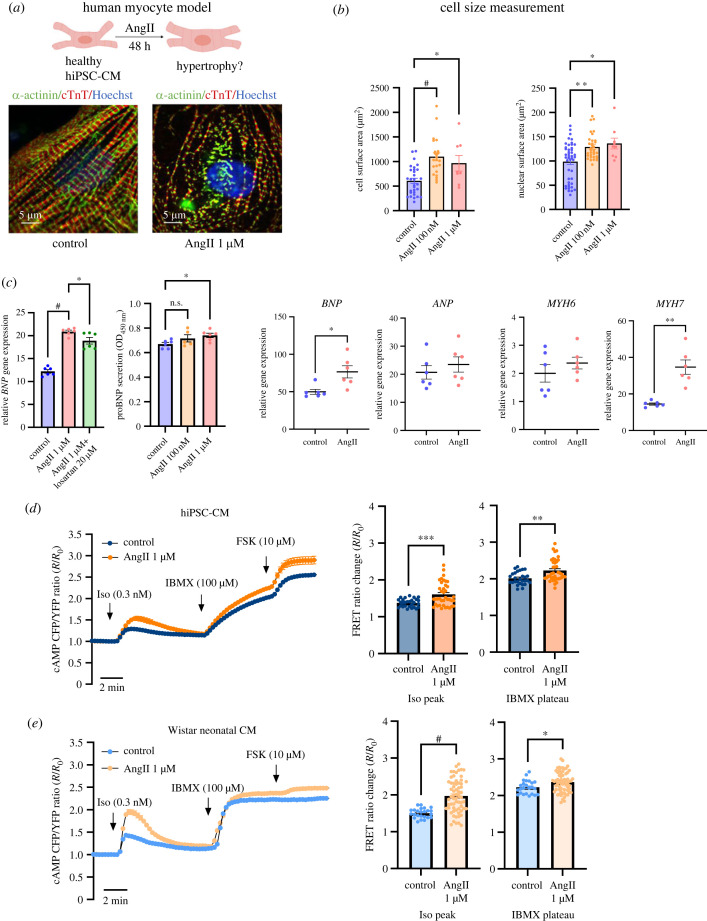
Hypertrophic phenotype induced by angiotensin II (AngII) in hiPSC-CM (from one differentiation batch of hiPSC SFC854-03-02) versus Wistar neonatal cardiomyocytes. (*a*) The cartoon shows the induction of cardiac hypertrophy by exposing healthy hiPSC-CM to AngII for 48 h. Staining of α-actinin and cTnT shows disruption of cardiac structural protein by 1 µM AngII. Sarcomeres were fragmented and not organized into parallel myofilament bundles. (*b*) Treatment with AngII resulted in significant increase of cell size (left) and nuclear size (right). (*c*) Brain natriuretic peptide (BNP) mRNA expression significantly increased in 1 µM AngII-treated myocytes. Pretreatment of myocytes with AngII receptor blocker losartan antagonized *BNP* enhancement. Quantitative ELISA in cell culture supernatant revealed a tendency for increased proBNP secretion in hypertrophic groups. RT-qPCR repeated with iPS-OX1-19 hiPSC-CM confirmed significant increase of *BNP* and *MYH7* (for myosin heavy chain 7), and an increased tendency for *ANP* and *MYH6*. (*d*,*e*) Grouped data showing dynamic increases in cAMP in response to isoprenaline (Iso), IBMX and forskolin (FSK) in hiPSC-CM (*d*) and Wistar neonatal cardiomyocyte (*e*) models. One micromolar Ang II-treated group caused beta-adrenergic hyper-responsiveness and increased maximal cAMP level in both models.

To develop myocyte hypertrophy, healthy hiPSC-CM (derived from SFC854-03-02 hiPSC) were exposed to AngII (100 nM and 1 µM) for 48 h. Myofibril structure of hypertrophic cardiac myocytes was distorted, as represented by discrete immunofluorescence staining of α-actinin and cTnT (figure 2*a*). Myofilament rearrangement is an indicator of stress-induced pathological hypertrophy. To assess cell enlargement, cell size and nuclear size were measured by immunostaining. Imaging fields for control and AngII-treated myocytes were randomly selected, all cells and nuclei with clear and complete borders in the view were used for surface area measurements to avoid selection bias. Cell surface area significantly increased from 606.3 ± 53.02 µm^2^ (control) to 1096 ± 79.89 µm^2^ (AngII 100 nM) and 964.7 ± 161.0 µm^2^ (AngII 1 µM), *p* < 0.01. Nuclei were significantly enlarged, from 98.46 ± 5.879 µm^2^ (control) to 135.6 ± 5.63 µm^2^ (AngII 100 nM) and 135.9 ± 11.0 µm^2^ (AngII 1 µM), *p* < 0.01. This is consistent with activated biosynthesis in the nucleus and enhanced protein synthesis in the cytosol (figure 2*b*; electronic supplementary material, data S1). Fetal gene reactivation is well documented in hypertrophic cardiac myocytes in the process of cardiac remodelling. Therefore, we performed qPCR to ascertain the RNA expression of *BNP* as one of these fetal genes in hiPSC-CM. *BNP* transcription in 1 µM AngII-treated myocytes was significantly higher (*p* < 0.01) than in the untreated control group. Co-incubation with the AngII receptor blocker losartan (20 µM) reversed the increase in *BNP* expression (figure 2*c*; electronic supplementary material, data S2). The proBNP secretion level measured by ELISA also showed a dose-dependent increase in cell culture medium of hypertrophic hiPSC-CM (figure 2*c*; electronic supplementary material, data S2). Reactivation of *BNP* expression was confirmed by iPS-OX1-19 hiPSC differentiated cardiomyocytes, along with another important cardiac hypertrophic marker *MYH7* (figure 2*c*; electronic supplementary material, data S2).

Elevated cAMP level during beta-adrenergic stimulation is commonly associated with hypertrophied myocytes [36,37]. hiPSC-CM loaded with the cAMP FRET-sensor Epac-S^H187^ were sequentially stimulated by 0.3 nM isoprenaline, 100 µM IBMX and 10 µM FSK. Cytosol cAMP generation during beta-receptor activation was significantly higher in AngII (1 µM)-treated iPSC-CM (1.377 ± 0.021, *n* = 28 versus 1.607 ±0.051, *n* = 39, *p* < 0.001). The non-selective phosphodiesterase inhibitor IBMX further increased cytosolic cAMP levels. To demonstrate the dynamic operating range of the sensor, FSK (10 µM) was added to saturate cytosolic cAMP at the end of experiments. Both cAMP elevations were more prominent in hypertrophic myocytes (figure 2*d*,*e*; electronic supplementary material, data S3). Similarly, the FRET response of hiPSC-CM closely resembled the cAMP response of hypertrophied neonatal rat ventricular cardiomyocytes when stimulated with isoprenaline, indicating a conserved phenotypic response across model systems (figure 2*d*,*e*; electronic supplementary material, data S3).

### (c) Functional connection between hiPSC-SN and hiPSC-CM in co-culture

**Figure 3.**
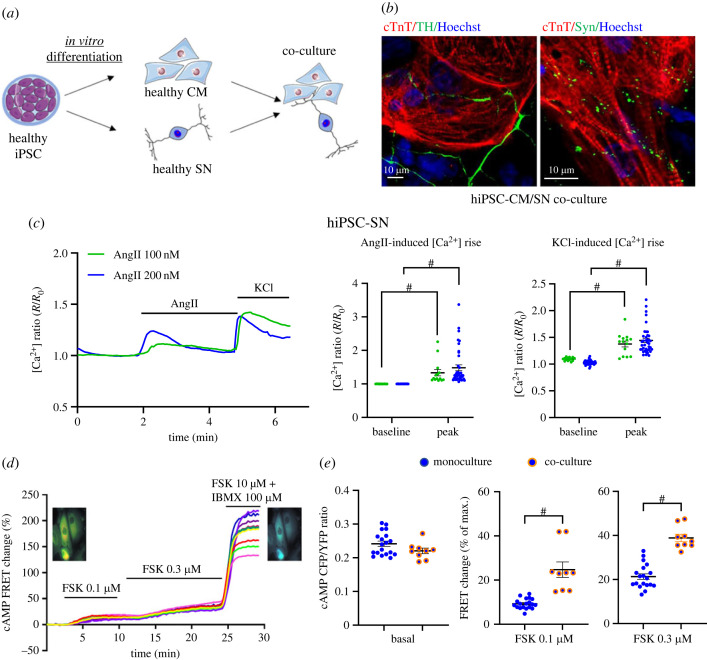
Functional phenotype of hiPSC-SN monoculture, hiPSC-CM monoculture and neurocardiac co-culture. (*a*) Cartoon showing cardiac myocytes and sympathetic neurons differentiated from a healthy hiPSC line and co-cultured together. (*b*) hiPSC-derived neurocardiac co-culture fixed and stained with cTnT (CM) and TH (SN) (left), cTnT (CM) and Syn (SN) (right). Overlay shows sympathetic neurons dispersed around cardiac myocyte clusters and extended axons towards myocytes, and dot-staining of anti-synapsin shows varicosities. (*c*) Representative normalized intracellular calcium ([Ca^2+^]_i_) trace of healthy hiPSC-SN monoculture (ReBl-PAT^RYR2-WT^). [Ca^2+^]_i_ level increased significantly at the application of 100 or 200 nM angiotensin II (AngII) and 50 mM KCl. (*d*) Original Förster resonance energy transfer (FRET) traces showing dynamics of cytosolic cAMP changes in response to adenylyl cyclase activator forskolin (FSK, 0.1 and 0.3 µM) in healthy 20-day iPSC-CM. Saturation of the sensor was achieved by using FSK (10 µM) and IBMX (100 µM). (*e*) Comparison of myocyte cytosolic cAMP changes between iPSC-CM monoculture and co-culture with iPSC-SN. Baseline cAMP level showed no significant difference between monoculture and co-culture, while co-culture with SN activated myocytes' response to FSK with significantly higher increase of cytosolic cAMP. ((*d*,*e*) from cell line iPS-OX1-19.)

To test whether myocytes and neurons differentiated from the same cell line could be a reliable *in vitro* model for the study of neurocardiac disease, we plated mature hiPSC-CM and hiPSC-SN in co-culture to establish neurocardiac connectivity (figure 3*a*). Re-plated hiPSC-SN extended axons toward hiPSC-CM and formed varicosities in close relation to myocytes, which indicated the formation of neurocardiac junctions. Staining with cTnT and TH showed the co-existence and connection of cardiac myocytes and sympathetic neurons in co-culture. Anti-synapsin staining showed the formation of varicosities around cardiac myocytes. Cardiac myocytes joined adjacent myocytes, forming clusters. Sympathetic neurons dispersed around cardiac myocytes clusters and axons stretched out to form connections with myocytes and other neurons (figure 3*b*) in a similar manner to early reports [35].

Prior to evaluation of sympathetic regulation of cardiac myocytes in hiPSC-derived co-culture, functional tests were first performed in hiPSC-SN monocultures. [Ca^2+^]_i_ was measured in hiPSC-SN derived from the healthy cell line ReBl-PAT^RYR2-WT^ using the calcium-sensitive fluorescent ratiometric dye Fura-2 AM. hiPSC-SN were bathed in Tyrode solution to reach a steady basal Ca^2+^ level, then stimulated by AngII (100 or 200 nM) followed by 50 mM KCl. [Ca^2+^]_i_ showed significant elevation from baseline level during the application of AngII (figure 3*c*; electronic supplementary material, data S4; *p* < 0.05) given the well-established presence of AT1 receptors on the neurons. Depolarization of neurons with 50 mM KCl also induced an abrupt [Ca^2+^]_i_ increase from 1.09 ± 0.01 to 1.38 ± 0.05, *p* < 0.0001 (in 100 nM AngII), and 1.03 ± 0.01 to 1.44 ± 0.04, *p* < 0.0001 (in 200 nM AngII) (figure 3*c*; electronic supplementary material, data S4). These results are consistent with the behaviour of neurons in response to receptor and voltage activation in the regulation of [Ca^2+^]_i_.

Subsequently, we recorded cAMP FRET changes in hiPSC-CM in monoculture and co-culture with hiPSC-SN in response to two concentrations of FSK to verify the functional neurocardiac connection (figure 3*d*; electronic supplementary material, data S5). Original traces showed increased cAMP generation in hiPSC-CM in response to FSK. Cytosolic cAMP at baseline showed no significant difference between hiPSC-CM in monoculture and co-culture. However, when stimulated by FSK (0.1 and 0.3 µM), the increase of cAMP was significantly higher in myocytes co-cultured with hiPSC-SN (*n* = 9) than monocultured myocytes alone (*n* = 19) (FSK 0.1 µM: 24.73 ± 3.49% versus 9.30 ± 0.53%, *p* < 0.0001; FSK 0.3 µM: 38.87 ± 1.71% versus 21.33 ± 1.26%, *p* < 0.0001). The greater cAMP response of the myocytes in the co-culture (compared with myocyte monoculture) is probably a combination of both FSK-activated myocyte cAMP, and the likely FSK-activated cAMP in neurons enhancing transmission to further increase cAMP in myocytes via beta-receptor activation of the second messenger pathway.

## 4. Discussion

Here, we present a differentiation protocol for the induction of sympathetic neurons and for generation of cardiac myocytes from two human iPSC cell lines. Both myocytes and neurons expressed cell-type-specific structural proteins and membrane receptors that underpin specific physiological function in response to stimuli. Derived myocytes were also capable of replicating pathological hypertrophy when challenged with AngII. Following calcium and cAMP phenotyping of monoculture hiPSC-SN and hiPSC-CM, we established hiPSC neurocardiac co-cultures and demonstrated anatomical and functional connectivity.

### (a) hiPSC cardiac myocyte differentiation and characterization

In the past decades, many protocols have been established for the differentiation of cardiac myocytes from hiPSC [8,9,31,38]. In this study, we adapted a widely used defined RPMI/B27 medium monolayer small-molecule-guided differentiation protocol [10]. The Wnt signalling pathway has been shown to have a biphasic effect in directing cardiogenesis. Early exposure to the GSK3 inhibitor CHIR99021 promotes the induction of mesoderm progenitor cells. Moreover, subsequent inhibition of canonical Wnt signalling by Wnt-C59 results in further differentiation to cardiac fate [8]. Indeed, the concentration of these small molecules influences the induction proficiency across different cell lines [8]. Here we used 6 µM CHIR99021 for 48 h (days 0–2) and 2.5 µM Wnt-C59 for 48 h (days 3–5), yielding *ca* 80% cTnT and MLC2v double-positive cardiac myocytes from two hiPSC lines. In addition, we found that hiPSC confluency when commencing cardiomyocyte differentiation is critical in optimizing cell yield. A high confluency of 90% in a 12-well plate was optimal in generating cardiomyocytes with spontaneous contraction from days 7–9 following induction. We also observed that derived myocytes were best purified by shifting the nutritional supply from glucose to lactate using RPMI-glucose medium supplemented with 5 mM sodium lactate from day 11 to day 14. This helped minimize non-cardiac cell proliferation over cardiac myocyte maturation since other cell types required glucose for differentiation and are intolerant to lactate metabolism, as reported by Gaspar *et al*. [39]. Therefore, we conducted experiments at around day 20 to avoid over-expansion of other cell types. For longer cell maintenance, medium could be changed to low-glucose maturation medium supplemented with fatty acids to minimize non-cardiac cell enhancement [40].

Pressure and volume overload and inherited mutations are common causes of pathological cardiac hypertrophy. For *in vitro* modelling, isoprenaline, adrenaline or noradrenaline mimics neurohormonal activation during pressure and stress overload. Agonists for AngII type I receptors and endothelin-1 receptors are all effective inducers for cardiac hypertrophy [41]. To characterize pathophysiological function of hiPSC-CM, these cells hypertrophied following incubation with 100 nM or 1 µM AngII for 48 h. We observed significant increases in cell size and nuclear enlargement with sarcomere disarray. In addition, expression of the fetal gene *BNP*, which is an established marker of cardiac hypertrophy, was also elevated. When stimulated by isoprenaline, cAMP levels were significantly enhanced in hypertrophic hiPSC-CM, which indicates remodelling of beta-receptor-coupled cAMP signalling. This adrenergic hyper-responsiveness was also replicated in neonatal Wistar rat ventricular myocytes, thus supporting the idea that the signalling pathway is conserved across mammalian systems.

### (b) hiPSC sympathetic neurons

Coupling of sympathetic neurons to cardiac myocytes is an emerging field to study the role of neuronal-myocyte signalling systems in a dish [22,42], although there are only a few studies that have investigated this coupling using human iPSC cell models [21,23,24]. We differentiated sympathetic neurons from hiPSC based on the published technique from Winbo *et al*. [24]. Since no standardized sympathetic neuronal induction protocol is available to date, our data serve as another validation for this feeder-free, chemical-directed induction process. We found that hiPSC-SN showed typical round cell bodies with extensive axonal outgrowth (figures 1*f* and 3*b*). Staining of TH and PRPH confirmed these neuron-like cells as catecholaminergic in nature, underscoring their being sympathetic neurons. Their functional electrophysiological properties, including resting membrane potential and action potential kinetics, have been described previously [24].

We further phenotyped these cells and tested their [Ca^2+^]_i_ in response to cell stimulation, since Ca^2+^ is a crucial messenger that underpins synaptic plasticity [43] and neurotransmission [44]. Using live cell calcium imaging we observed that [Ca^2+^]_i_ could be enhanced in response to AngII and high extracellular potassium. AngII acts through AT1 receptors, which activate phospholipase C, which cleaves the phospholipid phosphatidylinositol 4,5-bisphosphate (PIP2) into diacyl glycerol (DAG) and inositol 1,4,5-trisphosphate (IP3). IP3 then diffuses in the cytosol and binds to IP3 receptors on the endoplasmic reticulum, which in turn releases stored Ca^2+^ into the cytosol [45]. High potassium-induced depolarization activates plasma membrane voltage-gated calcium channels, which allows extracellular Ca^2+^ influx to activate downstream signalling pathways involved in exocytosis of classical transmitters [46]. In particular, Winbo *et al*. [26] demonstrated the utility of this neuronal monoculture system when sympathetic neurons derived from an LQT1 lineage had increased excitability and transmission, suggesting that a component of the disease phenotype may also reside in the neuron [26]. When taken together with those of Winbo *et al.*, our observations are consistent with the notion that our neuronal hiPSC are sympathetic in nature and can respond to classical physiological stimuli.

### (c) Neural-cardiac signalling in co-culture

Several groups have successfully coupled healthy iPSC sympathetic neurons and cardiac myocytes and shown the power of the neuron to drive cardiac excitability [21,23,24]. We have further demonstrated the utility of the hiPSC neurocardiac co-culture system. First, both neurons and myocytes were able to survive and thrive in co-culture. Secondly, immunostaining confirmed the formation of neuronal varicosities adjacent to myocyte clusters, which is the anatomical basis for functional connectivity. Physiologically, the increased cytosolic cAMP response to adenylyl cyclase activation was found to be enhanced in myocytes co-cultured with neurons compared with myocyte monocultures. The augmented enzyme function could be a result of myocyte maturation when co-culturing with neurons [25,47], where recent studies suggest secreted neurotrophic factors enhance the synaptic efficacy of the co-culture [42,48,49]. Our co-culture responded in a dynamic fashion to activation of cAMP with FSK, resulting in a larger FRET signal in co-cultured myocytes compared with myocytes in monoculture. Here, FSK application to the co-culture would give rise to increased neurotransmission, which would act on cardiac myocytes as an extra stimulus to raise myocyte cAMP.

In conclusion, we have replicated various anatomical and physiological characteristics of human cells by hiPSC-CM and hiPSC-SN. Whether these cell types have the full transcriptomic atlas of actual human cells remains to be established. Nevertheless, the hiPSC monoculture and co-culture preparations behave in a manner that mimics key physiological responses seen in other mammalian cell types, and therefore may provide a framework for target discovery and a mechanistic understanding of human pathophysiological states.

## Data Availability

The datasets generated during and/or analysed during the current study are available as supplementary material [50].
